# Proportion of NAFLD patients with normal ALT value in overall NAFLD patients: a systematic review and meta-analysis

**DOI:** 10.1186/s12876-020-1165-z

**Published:** 2020-01-14

**Authors:** Xuefeng Ma, Shousheng Liu, Jie Zhang, Mengzhen Dong, Yifen Wang, Mengke Wang, Yongning Xin

**Affiliations:** 1 Department of Infectious Disease, Qingdao Municipal Hospital, Qingdao University, Qingdao, 266011 China; 2 Central Laboratories, Qingdao Municipal Hospital, Qingdao University, Qingdao, 266011 China; 3 Digestive Disease Key Laboratory of Qingdao, Qingdao, 266071 China

**Keywords:** Non-alcoholic fatty liver disease, Non-alcoholic steatohepatitis, ALT, Meta-analysis

## Abstract

**Background:**

ALT value is often used to reflect the hepatic inflammation and injury in NAFLD patients, but many studies proved that ALT values were normal in many NAFLD patients. The aim of this study was to identify the summarized proportion of NAFLD patients with normal ALT value in the overall NAFLD patients.

**Methods:**

Electronic databases PubMed, EMBASE, Ovid, and the Cochrane Library were searched for potential studies published from January 1, 2000 to September 30, 2019. Studies that have reported the number of NAFLD or NASH patients with normal and abnormal ALT value were included and analyzed. Abstracts, reviews, case reports, and letters were excluded.

**Results:**

A total of 11 studies with 4084 patients were included for assessing the summarized proportion of NAFLD patients with normal ALT in overall NAFLD patients. As the results shown, the summarized proportion of NAFLD patients with normal ALT value in overall NAFLD patients was 25% (95%CI: 20–31%) which was calculated by the random-effects model. The summarized proportion of NASH patients with normal ALT value in overall NASH patients was 19% (95%CI: 13–27%). Subgroup analysis includes region, study type, diagnostic method, and group size were conducted to investigate the resource of heterogeneity in the summarized proportion of NAFLD and NASH patients with normal ALT value.

**Conclusions:**

25% NAFLD patients and 19% NASH patients possess the normal ALT value in the clinical manifestation. The value of ALT in the clinical diagnosis of NAFLD and NASH remains need be further testified.

## Background

Non-alcoholic fatty liver disease (NAFLD) has become the most prevalent chronic liver disease with an estimated prevalence of 25–45% in western countries and 29.62% in Asia [1–4]. NAFLD is caused by the excessive intrahepatic fat deposition that without a specific cause such as excessive alcohol consumption, viral hepatitis, or a hereditary disorder [5, 6]. NAFLD disease spectrum ranges from simple steatosis to non-alcoholic steatohepatitis (NASH), fibrosis, cirrhosis and even the hepatocellular carcinoma (HCC) [7, 8]. Many metabolism-related diseases such as type 2 diabetes, insulin resistance, obesity, coronary artery disease and dyslipidemia are tightly correlated with NAFLD [9].

Alanine aminotransferase (ALT) is an enzyme which exists richly in the cytosol of hepatocytes. Usually, less ALT can be detected in the serum of health population, once the apoptosis and injury of hepatocytes were occurred, the ALT value in the serum increased significantly [10]. As a standard indicator of liver function, serum ALT value is usual used to reflect hepatic inflammation and liver injury in patients with various chronic liver diseases. Most of the time, only subjects who with increased ALT values were enrolled in the clinical investigation or trials. In most of the previous studies, the higher ALT values were tightly associated with the higher risk of NAFLD especially with the NASH [11, 12], but some other studies showed that NAFLD or NASH patients which measured by histology, MRI and ultrasonography possessed the normal ALT value [13–15]. Moreover, some studies showed that patients with normal ALT levels had the histological features of disease progression [16, 17]. NAFLD patients with normal ALT values were often neglected because most physicians evaluate the hepatic risk of NAFLD based on the change of ALT value.

Accumulated studies had been trying to identify the proportion of NAFLD patients with normal ALT value in the overall NAFLD patients and looking for the classical histological features of NAFLD patients with normal ALT value, but no consistent results were acquired. The aim of this study was to identify the summarized proportion of NAFLD patients with normal ALT value in the overall NAFLD patients of all the appropriate studies. To our knowledge, this is the first systematic review and meta-analysis that identifying the summarized proportion of NAFLD patients with normal ALT value in the overall NAFLD patients.

## Methods

### Search strategy

This study was performed according to the recommendations of the Moose [18]. Electronic databases include PubMed, EMBASE, Ovid, and Cochrane Library were searched to obtain all the potential appropriated studies from January 1, 2000 to September 30, 2019. The search keywords used in this study were as follows: NAFLD, Non-alcoholic fatty liver disease, NASH, non-alcoholic steatohepatitis, Normal Alanine Aminotransferase, and Normal ALT. We also manually searched the references of the selected articles to identify additional studies. Only English articles were included in this study.

### Inclusion and exclusion criteria

The initially retrieved publications were reviewed by two investigators (Xuefeng Ma and Shousheng Liu) independently. The discrepancy was resolved by discussion with all investigators. Studies that met the following criteria were included: 1) conducted the case-control, cohort study or cross-sectional analysis; 2) NAFLD was diagnosed by MRI or Ultrasonography and the NASH was diagnosed by histology; 3) ALT value was measured on the biochemical laboratory; 4) the number of NAFLD or NASH patients with normal and abnormal ALT was reported. Abstracts, reviews, case reports, and letters were excluded from this study. Studies that absence of measurement of the number of NAFLD or NASH patients with normal and abnormal ALT values were also excluded from this study.

### Quality assessment and data extraction

The quality of all the included studies was assessed independently by two authors (Xuefeng Ma and Shousheng Liu) using the Newcastle-Ottawa Scale (NOS) tool for case-control and cohort study [19]. We assigned the NOS scores of 1–3, 4–6, and 7–9 for low-, intermediate-, and high-quality studies. Cross-sectional analysis was not accessed by the NOS tool. The discrepancy was resolved by discussion with all the investigators. The following information were extracted from each study: first author, publication time, the sample size, country, the number of NAFLD or NASH patients with normal ALT, the number of total NAFLD or NASH patents. The data were collected independently by two investigators (Xuefeng Ma and Shousheng Liu).

### Statistical analysis and data synthesis

Random-effects model was used to estimate the pooled proportion of NAFLD patients with normal ALT value. Statistical heterogeneity among studies was assessed by Q and *I*^*2*^ statistics. For the Q statistic, heterogeneity was considered to be present when *P* <  0.1 or *I* ^*2*^ > 50%. Publication bias was evaluated visually by funnel plots and the publication bias was considered significant when *P* value was less than 0.05 in either Begg’s test. The subgroup was carried out by region, type of study, outcome measurement and group size. We used the metaphor (version 2.0–0) and meta (version 4.9–5) packages of R (version 3.6.1) to conduct the different analyses [20] and all statistical tests.

## Results

### Study characteristics

**Fig. 1:**
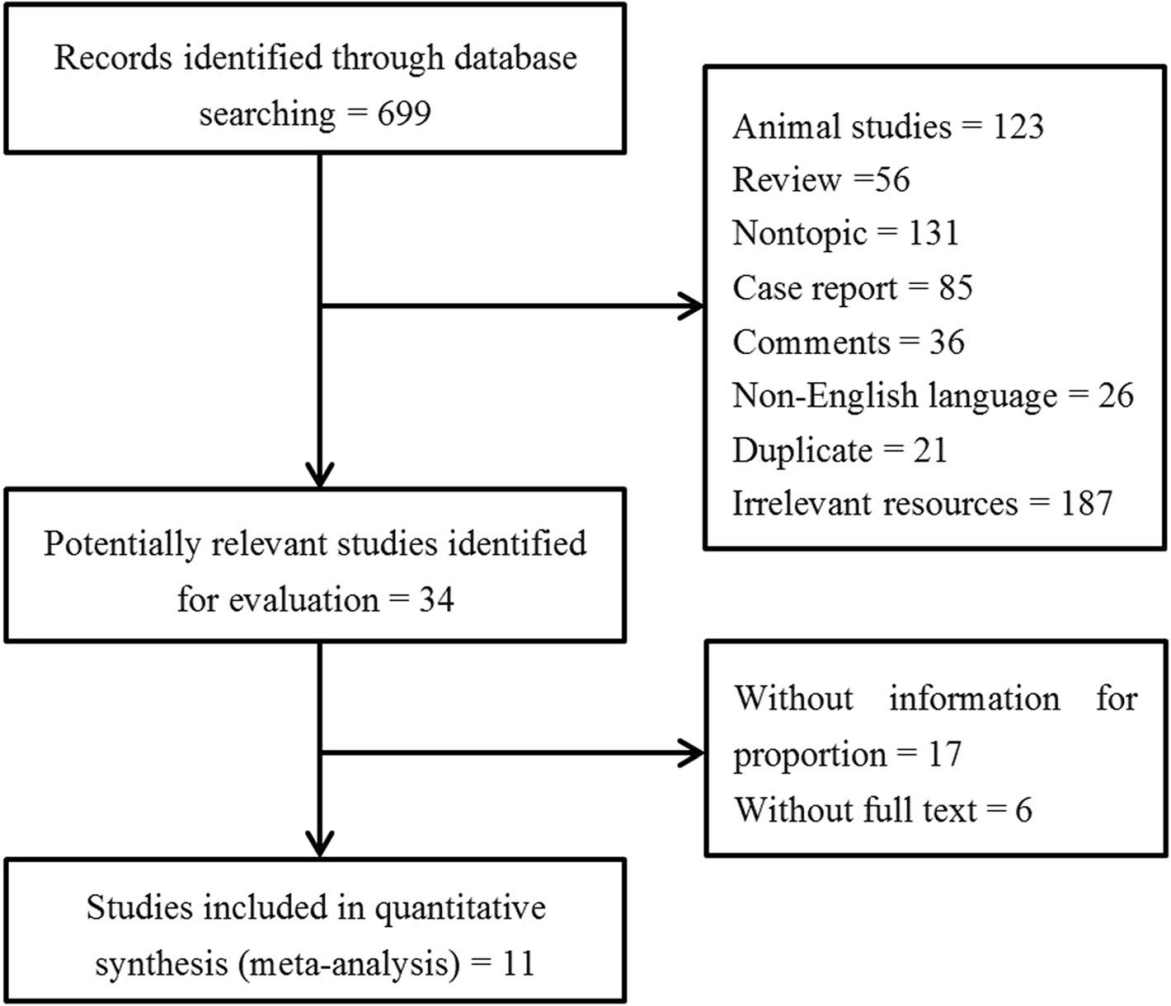
Flow chart of the literature search process

The flow diagram of studies selection was shown in the Fig. 1. A total of 699 references were identified according to our search strategy. After removed the duplication, reviews, animal studies, and irrelevant resources, 34 potential studies were selected to further evaluation. After excluded 23 improper studies, a total of 11 studies with 4084 patients [13–15, 21–28] which matched the inclusion criteria were included in this meta-analysis.

The main features of the included studies were shown in the Tables 1, which including 6 retrospective cohort studies, 3 prospective cohort studies and 2 cross-sectional analyses. Among these studies, subjects in 6 studies were NASH patients. Among all of the included studies, 4 studies are from North America, 3 from Europe and 4 from Asia. NOS scores suggested that 9 studies possessed the high quality with all the NOS scores were 7. The two cross-sectional studies were not assessed.

**Table 1:** Features of the 11 studies included in this meta-analysis

Study	Type of Study	Country	NAFLD patients with normal ALT (n)	Overall patients (n)	Diagnostic methods	NOS score
Mofrad et al.,2003^21^	Retrospective	USA	51	386	Histology	7
Amarapurkar et al.,2004^22^	Prospective	India	25	81	Histology	7
Fracanzani et al.,2008^23^	Retrospective	Italy	63	458	Histology	7
Uslusoy et al.,2009^24^	Retrospective	Turkey	9	34	Histology	7
Wong et al.,2009^25^	Prospective	HongKong (China)	38	173	Histology	7
Verma et al.,2013^26^	Retrospective	USA	56	222	Histology	7
McPherson et al.,2013^27^	Retrospective	UK	70	305	Histology	7
Maximos et al.,2015^28^	Prospective	USA	165	380	MRI	7
Umehara et al.,2018^14^	Cross-sectional	USA	353	1616	Ultrasonography	NA
Sheng et al.,2018^13^	Retrospective	China	69	212	Ultrasonography	7
Sun et al.,2019^15^	Cross-sectional	China	75	217	Histology	NA

Abbreviation: *CI* Confidence interval

### Proportion of NAFLD patients with normal ALT in overall NAFLD patients

**Fig. 2:**
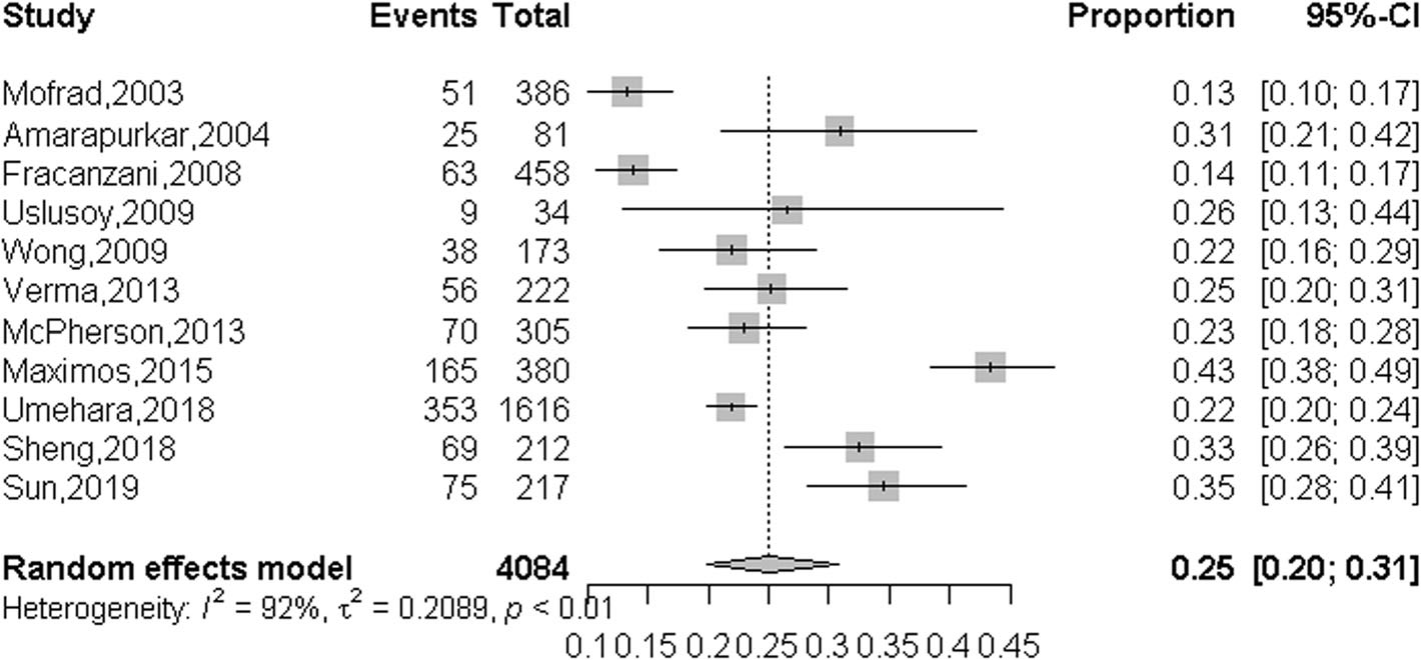
Summarized proportion of NAFLD patients with normal ALT value in overall NAFLD patients

To investigate the pooled proportion of NAFLD patients with normal ALT in overall NAFDL patients, 11 studies from different countries and regions which include 4 from the USA, 1 from Italy, 1 from India, 1 from Turkey, 1 from the UK, and 3 from China were included. The overall pooled proportion were 0.25 (95%CI: 0.20–0.31) which calculated by random-effects model (*P* <  0.001, *I*^*2*^ = 92.0%) (Fig. 2).

**Fig. 3:**
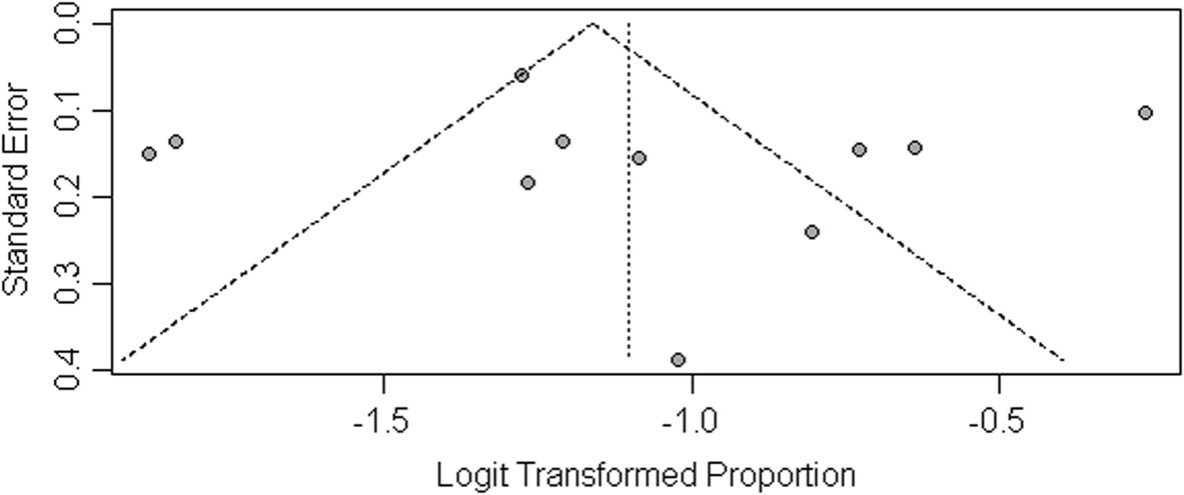
Funnel plot of publication bias on the proportion of NAFLD patients with normal ALT value

Subgroup analysis was performed to explore the sources of heterogeneity. We evaluated potential sources of heterogeneity between region, type of study, diagnostic methods and group size (Table 2). The summarized proportion of NAFLD patients with normal ALT value in Asia was 0.30 (95%CI: 0.25–0.35, *I*^*2*^ = 52.0%), which higher than in North America 0.24 (95%CI: 0.16–0.36, *I*^*2*^ = 97.0%) and Europe 0.19 (95%CI: 0.14–0.26, *I*^*2*^ = 72.0%). The summarized proportion of NAFLD patients with normal ALT value in prospective cohort study group was 0.32 (95%CI: 0.22–0.43, *I*^*2*^ = 85.0%), which higher than in retrospective cohort study group 0.21 (95%CI: 0.10–0.28, *I*^*2*^ = 87.0%) and cross-sectional analysis group 0.27 (95%CI: 0.19–0.37, *I*^*2*^ = 85.0%). The summarized proportion of NAFLD patients with normal ALT value in MRI diagnostic group was 0.43 (95%CI: 0.39–0.38), which higher than in the Histology diagnostic group 0.22 (95%CI: 0.17–0.28, *I*^*2*^ = 85.0%) and Ultrasonography diagnostic group 0.26 (95%CI: 0.20–0.34, *I*^*2*^ = 83.0%). The summarized proportion of NAFLD patients with normal ALT value in more than 300 size group was 0.27 (95%CI: 0.23–0.33, *I*^*2*^ = 97.0%), which higher than in less than 300 size group 0.22 (95%CI: 0.14–0.31, *I*^*2*^ = 46.0%). Funnel plots was constructed to investigate the publication bias, and the results suggested that no publication bias was exist (*P* = 0.14) (Fig. 3).

**Table 2:** Subgroup meta-analysis by region, type of study, diagnostic method and group size for the summarized proportion of NAFLD patients with normal ALT in overall NAFLD patients

Variable	Summarized proportion	95% CI	*I*^*2*^	*P* value	Number of studies
Overall estimate	0.24	[0.19; 0.30]	92%	< 0.01	11
Region					
North America	0.24	[0.16; 0.36]	97%	< 0.01	4
Asia	0.30	[0.25; 0.35]	52%	0.05	4
Europe	0.19	[0.14; 0.26]	72%	< 0.01	3
Type of study					
Retrospective cohort study	0.21	[0.16; 0.28]	87%	< 0.01	6
Prospective cohort study	0.32	[0.22; 0.43]	85%	< 0.01	3
Cross-sectional analysis	0.27	[0.19; 0.37]	85%	< 0.01	2
Diagnostic method					
Histology	0.22	[0.17; 0.28]	85%	< 0.01	8
MRI	0.43	[0.39; 0.38]	NA	NA	1
Ultrasonography	0.26	[0.20; 0.34]	83%	< 0.01	2
Group size					
More than 300	0.22	[0.14; 0.31]	97%	< 0.01	5
300 or less than 300	0.27	[0.23; 0.33]	46%	0.06	6

Abbreviation: *CI* Confidence interval

Sensitivity analysis was carried out to evaluate the influence of a single study on the results of this meta-analysis. We found that no significant changed was observed of *I*^*2*^ values when anyone study was removed from this meta-analysis.

### Summarized proportion of NASH patients with normal ALT in overall NASH patients

**Fig. 4:**
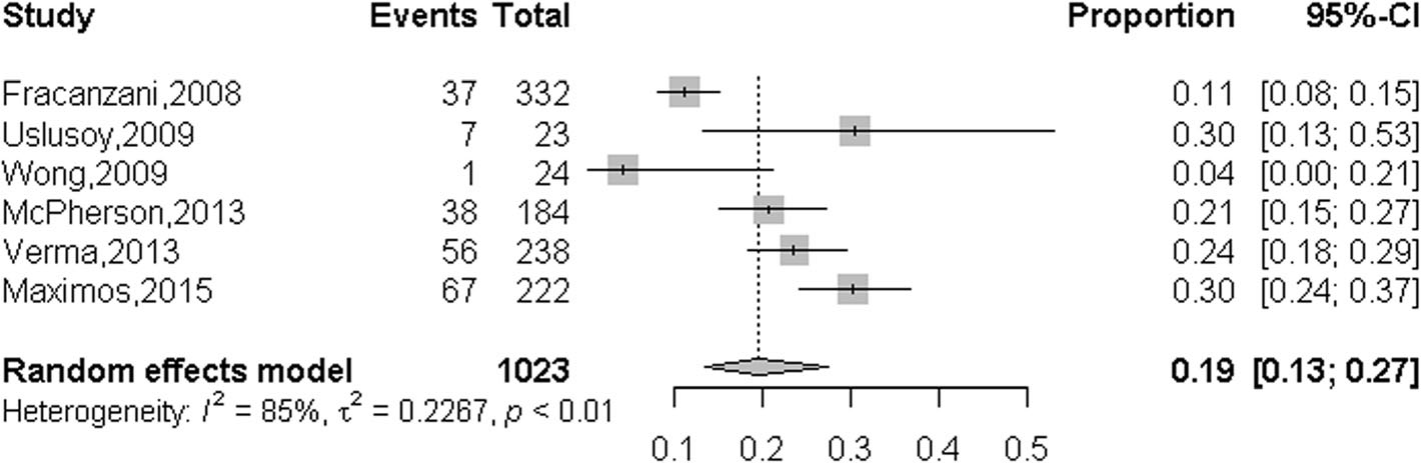
Summarized proportion of NASH patients with normal ALT value in overall NASH patients

6 of the 11 studies with 1023 patients were selected for the meta-analysis of the summarized proportion of NASH patients with normal ALT in overall NASH patients [23–28]. As shown in the Fig. 4, the summarized proportion NASH patients with normal ALT in overall NASH patients was 19% (95%CI: 13–27%), which calculated by the random-effects model (*P* <  0.001, *I*^*2*^ = 85.0%).

The subgroup analysis was used to explore the sources of heterogeneity. We evaluated the possible sources of heterogeneity between studies, including region, type of study, diagnosis method and group size (Table 3). The summarized proportion of NASH patients with normal ALT value in North America was 0.25 (95%CI: 0.19–0.33), which higher than in Asia 0.04 (95%CI: 0.01–0.24, *I*^*2*^ = 85.0%) and Europe 0.19 (95%CI: 0.11–0.30, *I*^*2*^ = 81.0%). The summarized proportion of NASH patients with normal ALT value in retrospective cohort study group 0.21 (95%CI: 015–0.29, *I*^*2*^ = 84.0%), which higher than in prospective cohort study group was 0.04 (95%CI: 0.00–0.21). The summarized proportion of NASH patients with normal ALT value in MRI diagnostic group was 0.30 (95%CI: 0.2–0.37), which higher than in histology diagnostic group 0.17 (95%CI: 0.12–0.25, *I*^*2*^ = 77.0%). The summarized proportion of NASH patients with normal ALT value in more than 300 size group was 0.20 (95%CI: 0.12–0.32, *I*^*2*^ = 91.0%), which equal to the proportion in less than 300 size group 0.20 (95%CI: 0.15–0.26, *I*^*2*^ = 0%).

**Table 3:** Subgroup meta-analysis by region, type of study, diagnostic method and group size for the summarized proportion of NASH patients with normal ALT in overall NASH patients

Variable	Summarized proportion	95% CI	*I*^*2*^	*P* value	Number of studies
Overall estimate	0.30	[0.24;0.37]	85%	< 0.01	6
Region					
North America	0.25	[0.19;0.33]	85%	< 0.01	3
Asia	0.04	[0.01;0.24]	NA	NA	1
Europe	0.19	[0.11;0.30]	81%	< 0.01	2
Type of study					
Retrospective cohort study	0.21	[0.15;0.29]	84%	< 0.01	5
Prospective cohort study	0.04	[0.00;0.21]	NA	NA	1
Diagnostic method					
Histology	0.17	[0.12;0.25]	77%	< 0.01	5
MRI	0.30	[0.25;0.37]	NA	NA	1
Group size					
More than 300	0.20	[0.12;0.32]	91%	< 0.01	3
300 or less than 300	0.20	[0.15;0.26]	0%	0.11	3

### Characteristics of NAFLD patients and NASH patients with normal ALT value

The characteristics of NAFLD patients and NASH patients with normal ALT value were analyzed. The results showed that the NAFLD patients with normal ALT value possess a tightly relationship with diabetes (OR = 2.30, 95% CI: 1.38–3.82; *P* <  0.01), hypertension (OR = 2.03, 95% CI: 1.47–2.80; *P* <  0.56) and metabolic syndrome (OR = 1.42, 95% CI: 1.00–2.00; *P* = 0.60). Furthermore, normal ALT was associated with the gender (male vs female; OR = 0.73, 95% CI: 0.40–1.32; *P* <  0.01), which suggested that female NAFLD patients are more prone to have the normal ALT value (Table 4). In the aspect of liver histology, the results indicated that normal ALT was related to steatosis grade (1 vs 2–3; OR = 4.30, 95% CI: 2.35–7.87; *P* = 0.10) and lobular inflammation (0–1 vs 2–3; OR = 3.35, 95% CI: 1.52–7.34; *P* = 0.36) in NAFLD patients (Table 4). In addition, Fracanzani et al. reported that HOMA-IR was tightly associated with the normal ALT value in NASH patients (OR = 1.9, 95% CI: 1.2–3.5 unit increase, *P* = 0.008) [23]. Verma et al. found that steatosis and ballooning were significant associated with the normal ALT value, but only the ballooning was significant increased in NASH patients with elevated ALT value which diagnosed by histology [24]. However, Uslusoy et al. demonstrated that there were no significant differences of clinical characteristics between the NASH patients with elevated ALT group and normal ALT value [27].

**Table 4:** Characteristics of NAFLD patients with normal ALT value

Characteristics	Number of studies	OR (95% CI)	*P* value	Heterogeneity	Effects model
Gender (male vs. female)	10	0.73 (0.40;1.32)	< 0.01	90%	Random
Diabetes	6	2.30 (1.38;3.82)	< 0.01	73%	Random
Hypertension	5	2.03 (1.47;2.80)	0.56	0%	Fixed
Metabolic syndrome	4	1.42 (1.01;2.00)	0.60	0%	Fixed
Steatosis grade(1 vs 2–3)	3	4.30 (2.35;7.87)	0.10	57%	Random
Lobular inflammation(0–1 vs 2–3)	2	3.35 (1.52;7.34)	0.36	0%	Random

## Discussion

NAFLD is usually caused by the abnormal metabolism in patients and tightly associated with dyslipidemia, diabetes mellitus and obesity [5]. Abundant studies and clinical practices have proven that the liver enzyme levels such as ALT and so on were increased usually in the NAFLD patients, and these liver enzymes could be used as the diagnostic markers for the NAFLD at some extent [29–31]. However, not all the studies supported the above conclusion. Sheng et al. reported that some NAFLD patients possessed the normal ALT value, and the residual NAFLD patients had the elevated level of ALT [13]. Amarapurkar et al. reported that the histological and clinical manifestation of NAFLD and NASH patients with normal ALT value were not different with the NAFLD and NASH patients with elevated ALT value [22]. In consideration of these controversial reports and the inconsistent conclusion of ALT value in NAFLD patients, we conducted this meta-analysis to explore the proportion of NAFLD patients with normal ALT value in overall NAFLD patients.

In this meta-analysis, we included 4084 patients of 11 studies which were from different regions and study types. We found that the summarized proportion of NAFLD patients with normal ALT value in overall NAFLD patients was 25% (95%CI: 20–31%), In view of the high heterogeneity of this analysis (*I*^*2*^ = 92.0%), we performed the subgroup analysis which include region, type of study, diagnostic method, and group size to investigate the resource of heterogeneity. Unfortunately, almost all the heterogeneity of each group was higher than 50%. In addition, we also explore the proportion of NASH patients with normal ALT value in overall NASH patients, and the summarized proportion was 19% (95%CI: 13–27%). Similarly, the heterogeneity of this analysis in NASH patients was also very high (*I*^*2*^ = 85.0%). The results of subgroup analysis suggested that all the heterogeneity of each group was higher than 50%. The potential resource of heterogeneity of the summarized proportion of NAFLD patients and NASH patients with normal ALT value may contributed by the genetic factors and individual difference, detailed studies should be conducted to illuminate this phenomenon in the future.

Many physicians pay more attentions to the risk of NAFLD and NASH in these patients with elevated ALT levels, and patients with normal ALT levels were often neglected. Nevertheless, many studies demonstrated that ALT should not be a non-invasive biomarker. Mofrad et al. found that the mean steatosis (1.60 vs. 2.16, *P* <  0.04) and perisinusoidal fibrosis scores (0.35 vs. 0.9, *P* <  0.049) were lower in patients with the lower ALT levels versus patients with higher ALT, and a low normal ALT value does not correspondence to the freedom of underlying steatohepatitis with advanced fibrosis [21]. Verma et al. found that the AUROC of ALT level relating NASH and advanced fibrosis were 0.62 and 0.46, respectively. Indicated that ALT levels is not the optimal indicator to predict NASH and advanced fibrosis [27]. At present, the accurate diagnostic of NAFLD and NASH should remain is the biopsy, although the serum biomarker such as ALT level could be as a significant reference, but it should not be regarded as the diagnostic standard for the NAFLD and NASH [24, 26, 27].

Our study had several limitations. Firstly, considerable heterogeneity among studies limits the reliability of the results. Although we performed subgroup and meta-regression analyses to investigate some potential sources of heterogeneity, the high levels of heterogeneity cannot be reasonably explained. Secondly, the included studies might exist the selection and recall biases. Finally, only 11 studies were included in this meta-analysis, not all the regions and countries were covered.

## Conclusions

In summary, we investigated the summarized proportion of NAFLD patients with normal ALT value in overall NAFLD patients. We found that 25% NAFLD patients and 19% NASH patients had the normal ALT values in the overall NAFLD and NASH patients. ALT value as a significant metabolic indicator did not possess the enough accuracy to diagnostic the NAFLD and NASH, liver biopsy is remains necessary to diagnosis the NAFLD and NASH accurately.

## Data Availability

All data generated or analyzed in this study are available from the corresponding author for the reasonable request.

## Abbreviations

ALT: Alanine aminotransferase
AUROC: Area under the receiver operating characteristic curve
CI: Confidence interval
HCC: Hepatocellular carcinoma
NAFLD: Non-alcoholic fatty liver disease
NASH: Non-alcoholic steatohepatitis
NOS: Newcastle-Ottawa Scale
